# Moiré‐Driven Topological Transitions and Extreme Anisotropy in Elastic Metasurfaces

**DOI:** 10.1002/advs.202200181

**Published:** 2022-03-06

**Authors:** Simon Yves, Matheus Inguaggiato Nora Rosa, Yuning Guo, Mohit Gupta, Massimo Ruzzene, Andrea Alù

**Affiliations:** ^1^ Photonics Initiative Advanced Science Research Center City University of New York New York NY 10031 USA; ^2^ Department of Mechanical Engineering University of Colorado Boulder Boulder CO 80309 USA; ^3^ Physics Program Graduate Center City University of New York New York NY 10026 USA

**Keywords:** hyperbolic, metasurface, moiré materials, phononics, quasi‐periodicity, topological transitions, wave steering

## Abstract

The twist angle between a pair of stacked 2D materials has been recently shown to control remarkable phenomena, including the emergence of flat‐band superconductivity in twisted graphene bilayers, of higher‐order topological phases in twisted moiré superlattices, and of topological polaritons in twisted hyperbolic metasurfaces. These discoveries, at the foundations of the emergent field of twistronics, have so far been mostly limited to explorations in atomically thin condensed matter and photonic systems, with limitations on the degree of control over geometry and twist angle, and inherent challenges in the fabrication of carefully engineered stacked multilayers. Here, this work extends twistronics to widely reconfigurable macroscopic elastic metasurfaces consisting of LEGO pillar resonators. This work demonstrates highly tailored anisotropy over a single‐layer metasurface driven by variations in the twist angle between a pair of interleaved spatially modulated pillar lattices. The resulting quasi‐periodic moiré patterns support topological transitions in the isofrequency contours, leading to strong tunability of highly directional waves. The findings illustrate how the rich phenomena enabled by twistronics and moiré physics can be translated over a single‐layer metasurface platform, introducing a practical route toward the observation of extreme phenomena in a variety of wave systems, potentially applicable to both quantum and classical settings without multilayered fabrication requirements.

## Graphical abstract

Elastic metasurface is made of interleaved spatially modulated pillar lattices. The resulting quasi‐periodic moiré patterns support topological transitions in the isofrequency contours, leading to strong tunability of highly directional waves.

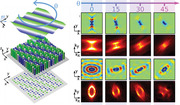

## 1 Introduction

New discoveries in condensed matter physics have recently shown how a twist in pairs of 2D stacked layers can produce highly unexpected emergent phenomena. Notably, the fine‐tuning of such twist allows the emergence of a magic angle at which a plethora of new phenomena can be observed, including flat‐band superconductivity,^[^ ^1^ ^]^ the quantum Hall effect,^[^ ^2^ ^]^ the creation of moiré excitons,^[^ ^3^, ^4^, ^5^, ^6^, ^7^, ^8^ ^]^ as well as interlayer magnetism.^[^ ^9^ ^]^ Based on these concepts, atomic photonic crystals in twisted bilayer graphene have shown the ability to route solitons^[^ ^10^, ^11^ ^]^ and produce quasi‐crystalline phases,^[^ ^12^ ^]^ higher‐order topology,^[^ ^13^ ^]^ non‐Abelian gauge potential,^[^ ^14^ ^]^ and helical topological state mosaics.^[^ ^15^, ^16^ ^]^ These phenomena, at the heart of the thriving field of twistronics,^[^ ^17^ ^]^ arise from the hybridization of the band structures associated with the two isolated monolayers, and the associated formation of moiré superlattices. Macroscopic‐scale implementations of these concepts using phononic and photonic metamaterials^[^ ^18^, ^19^ ^]^ have demonstrated flat bands in macroscopic analogues of bilayer graphene,^[^ ^20^, ^21^, ^22^, ^23^ ^]^ field localization within moiré lattices,^[^ ^24^, ^25^, ^26^ ^]^ the destruction of valley topological protection,^[^ ^27^ ^]^ artificial gauge fields,^[^ ^28^ ^]^ and broadband tunable bianisotropy for biosensing applications.^[^ ^29^, ^30^, ^31^ ^]^

These concepts have also been recently transposed to optical metamaterials, based on extreme anisotropic responses over hyperbolic metasurfaces (HMTs).^[^ ^32^ ^]^ Their iso‐frequency contours (IFCs) support an open, hyperbolic topology,^[^ ^33^, ^34^, ^35^, ^36^, ^37^ ^]^ featuring wave propagation with enhanced local density of states, and enabling subwavelength imaging, as well as negative refraction and canalization, inherently broadband in nature. By stacking two hyperbolic metasurfaces and rotating one with respect to the other, it is possible to largely modify the IFCs, inducing transitions between different topologies, from hyperbolic to elliptical.^[^ ^38^ ^]^ Such effect is the wave analogue of a Lifshitz transition in electronic band structures,^[^ ^39^ ^]^ which is known to play a crucial role in the physics of Weyl and Dirac semimetals.^[^ ^40^ ^]^ These exciting phenomena have also been recently demonstrated in polaritonic systems.^[^ ^41^, ^42^, ^43^ ^]^

The remarkable features of twisted bilayers exploit the interplay between two distinct layers with exotic wave responses, especially for field localization effects, and generally require a precise control over their coupling, alignment and twist angle. Hence, experimental setups, however reconfigurable, are quite challenging.^[^ ^44^ ^]^ In an attempt to circumvent such difficulties, a few recent studies have theoretically explored the emergence of analogous responses in single‐layer systems, with interactions or properties modulated by a second virtual layer. For instance, quasi‐flat bands, Dirac cones, and quantum anomalous phases have been predicted in modulated optical lattices,^[^ ^45^, ^46^ ^]^ while topological spectral gaps characterized by second Chern numbers akin to the 4D quantum Hall effect were illustrated in phononic lattices.^[^ ^47^ ^]^

Lifting the requirement of two stacked layers opens new prospects for the implementation of twistronics across several electronic, photonic, and phononic platforms. Toward this goal, in this Letter we explore the effects of emergent moiré patterns in monolayer pillared metasurfaces formed by the relative twist of two 2D spatial features: the lattice defined by the position of the pillars and the one defined by the anisotropic modulation profile of their height, which defines their resonant features. We first demonstrate that aligning these two lattices, resulting in an untwisted metasurface, and controlling their features, can produce a wide range of elliptical and hyperbolic IFCs. Next, we show that introducing a relative rotation between these two 2D lattices generates quasi‐periodic moiré patterns governing topological transitions between open and closed IFCs for specific twist angles. Such transitions inherently occur in a different way from those emerging in twisted bilayers,^[^ ^32^, ^41^, ^42^, ^43^ ^]^ for which the interplay between two material hyperbolic surfaces defines the transition instead of the emerging moiré patterns.We demonstrate the extreme wave phenomena in such twisted interleaved lattices with a highly reconfigurable metasurface formed by LEGO pillar‐cone resonators over an elastic plate, which is a 2D extension of previously employed implementations used to study the role of disorder^[^ ^48^ ^]^ and quasi‐periodicity.^[^ ^49^ ^]^ Our results show the great potential of this platform to study analogues of condensed matter phenomena at the macroscopic scale and in classical settings, and open the door to applications harnessing both strong anisotropy and moiré physics for enhanced wave manipulation.

## 2 Results and Discussion

**Figure 1:**
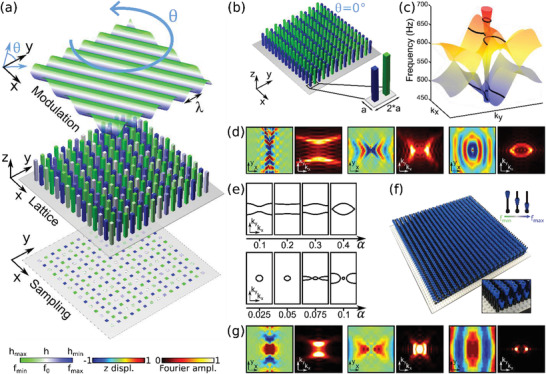
a) Moiré interleaved metasurface: A square lattice of pillars whose heights are modulated according to a rotating profile, here for *α* = 0.1. b) Schematic of the periodic system with $\theta \;=0^{\circ }\;$, and corresponding unit cell (inset). c) Numerical band structure of (b) with three contours corresponding to hyperbolic (at 485 Hz) along *y*, hyperbolic along *x* (at 625 Hz) and elliptical (at 670 Hz) highlighted with black lines. d) Simulated displacement field and corresponding spatial FT zoomed in at the center of the Brillouin zone for the three frequencies highlighted in (c). e) Modification of the IFCs as a function of height modulation at 485 Hz (top) and 625 Hz (bottom). f) Corresponding sample made of LEGO elements with cones at alternating heights (inset). g) Experimentally measured out‐of‐plane displacement field map and corresponding spatial FT at 345 Hz (left), 470 Hz (center), and 510 Hz (left).

Our metasurface consists of a thin elastic plate featuring an array of pillars in a square lattice of period *a* (**Figure** **1**a). The pillars can be modeled as mechanical dipolar resonators coupled to the transverse motion of the plate, and they are characterized by two bending resonant modes (along *x* and *y*) of equal frequency due to their symmetric cross section. Pillar‐type resonators have been employed in the design of metamaterials and metasurfaces, notably in the context of bandgaps,^[^ ^50^, ^51^, ^52^, ^53^, ^54^ ^]^ cloaking,^[^ ^55^ ^]^ and for seismic mitigation.^[^ ^56^ ^]^ Nonsymmetric embedded resonators have previously been used to implement elastic hyperbolic metamaterials,^[^ ^57^, ^58^, ^59^, ^60^, ^61^ ^]^ whereby large asymmetric couplings within the plate can generate anisotropic effective properties, which can be exploited in the context of waveguiding and subdiffraction imaging. Rather than breaking the resonator symmetry, here we induce strong anisotropy through lattice effects, by spatially modulating the resonant features of the array with a wavelength *λ* = *Na*. This effect introduces a spatial mismatch within the lattice, effectively creating a resonant macrocell including *N* distinct resonators responsible for asymmetric couplings across the metasurface. More specifically, we modify the pillar heights according to the modulation profile *S* (*x*, *y*, *θ*) = cos [2*π*/*λ*(cos *θx* + sin *θy*)], where *θ* is a twist angle measured with respect to the *x* axis (Figure 1a). The height *h_n_* of each pillar defines the resonant frequency of the dominant mode of interest, and it is assigned by sampling the modulation surface at the lattice sites *x_n_*, *y_n_*, i.e., *h_n_* = *h*_0_ [1 + *αS*(*x_n_*,*y_n_*,*θ*)], where *h*_0_ is the mean height and *α* is the modulation amplitude. This scheme generates two interleaved spatial features, consisting of the underlying square lattice of period *a* and of the sampled height distribution at the lattice sites.

We begin by highlighting the wave propagation features of the plate in the untwisted ($\theta \;=0^{\circ }\;$, *α* = 0.1) configuration, with period *a* along *y* and *Na* along *x*. We consider *N* = 2, resulting in a diatomic lattice of resonators (Figure 1b), for which the band structure is shown in Figure 1c (the complete band structure can be found in Figure S1, Supporting Information). All band structure computations and response simulations here are calculated with COMSOL Multiphysics, with details provided in the Section S1 (Supporting Information). The interleaving of the two lattices corresponding to the position of the resonators on the plate and to the resonance modulation, introduces an asymmetry and therefore a mismatch in IFCs along *x* and *y*, which results in hyperbolic IFCs around the resonance, two of which are highlighted by black lines in the figure, as well as elliptical ones. The existence of hyperbolic and elliptical IFCs is confirmed by simulating the harmonic response due to a point source excitation applied at the center of a finite sample comprising 80 × 80 unit cells. The resulting out‐of‐plane displacement field, and its Fourier transform (FT) displayed in Figure 1d, illustrate the emergence of hyperbolic and elliptic bands for the three frequencies marked in Figure 1c, namely 485 Hz, 625 Hz and 670 Hz. Modifying the height modulation, quantified by the parameter *α*, can dramatically change the coupling asymmetry within the surface, and correspondingly tailor the IFC shape. Two examples are displayed in Figure 1e at the frequencies of the hyperbolic contours in Figure 1c. In the top panel, at 485 Hz, an increase in *α* results in an inversion of IFC curvature, which changes from hyperbolic, to flat, to open‐elliptical, to finally close into an elliptical shape, demonstrating a topological transition. In the bottom panel, at 625 Hz, another topological transition from hyperbolic to elliptical phases occurs, this time as *α* decreases. In this case, the presence of elliptical IFCs at neighboring frequencies (Figure 1c) facilitates the transition between the two regimes, requiring smaller variations of *α* to drive the process.

We confirm these phenomena experimentally using our elastic metasurface platform, exploiting the fact that underlying sampling lattice is square. Our metasurface comprises 44 × 44 resonators whose heights are modulated with *λ* = 2*a* (Figure 1f), and we choose the maximum value of *α* allowed by the LEGO pillar geometry, as shown in the inset. The resonances are tuned by sliding the cones along the pillars, following the modulation of *h_n_* defined above (Figure 1f, inset). Our LEGO platform provides straightforward tunability and reconfigurability, which we harness to demonstrate extreme wave phenomena and topological transitions. The plate is excited at its center by an electrodynamic shaker, which applies a pseudo‐random excitation in the 200 − 700 Hz range, and the resulting out‐of‐plane wave fields are recorded by a scanning Doppler vibrometer (see Figure S2, Supporting Information for the experimental setup). While some hybridization exists between symmetric and asymmetric Lamb waves in the close vicinity of the resonances due to out‐of‐plane breaking of mirror symmetry, the modes of interest are mainly polarized along the out‐of‐plane direction (see Figure S3, Supporting Information for the out‐of‐plane polarization). Figure 1g displays the real and reciprocal space maps of the measured fields at three selected frequencies (345, 470, and 510 Hz): the measured hyperbolic and elliptical propagation are consistent with those predicted in simulations, with a small frequency shift attributed to minor differences between experimental and numerical models (see Figure S1, Supporting Information for the simulation of the LEGO lattice band structure). These results clearly show that a spatially anisotropic resonance frequency modulation can generate broadband hyperbolic mechanical Lamb waves, easily implemented over our platform. Moreover, the straightforward tuning of the height modulation amplitude enables a precise control and drastic variations of the supported IFCs.

**Figure 2:**
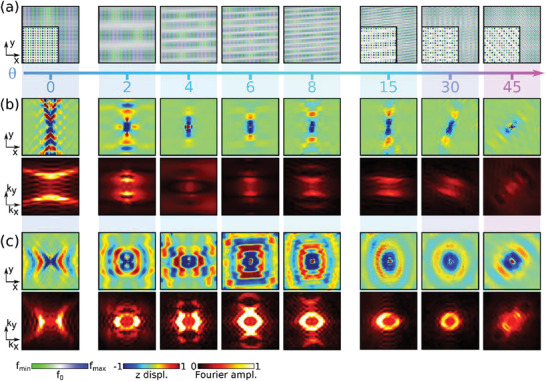
a) Pillar height modulation profile as a function of the rotation angle (zoomed detail in inset). b) Simulated out‐of‐plane displacement field maps (top) and spatial FT (bottom) as a function of the rotation angle for hyperbolicity along the *y* axis at 485 Hz. c) Same as (b) for the hyperbolicity along the *x* axis at 625 Hz.

Next, we explore the effect of rotating the interleaved lattices, by twisting the modulation profile relative to the underlying square lattice of resonators (Figure 1a). The misalignment between the lattice and modulation profile produces moiré patterns associated with complex spatial arrangements of the couplings. As illustrated in **Figure** **2**a, 2D modulation patterns with a strong angular dependence appear for $0^{\circ }<\theta <45^{\circ }$ (after $45^{\circ }$, the behavior is simply inverted because of symmetry). For a generic twist angle, the resulting pattern is quasiperiodic, and the periodicity is only restored for specific angles *θ* = cos ^−1^(*p*_2_/*q*), where {*p*_2_,*q*} are integers belonging to a Pythagorean triple satisfying $p_{1}^{2}+\;p_{2}^{2}=q^{2}$.^[^ ^24^ ^]^ These periodic configurations are characterized by unit cells that are typically very large: for instance, the two smallest super‐cells are obtained for *θ* = cos ^−1^(4/5)≅36.87°, resulting in a 5 × 10 super‐cell, and *θ* = cos ^−1^(12/13) ≅22.62°, resulting in a 13 × 26 super‐cell. The complexity of the periodic angles and the increasing size of the super‐cells makes the analysis through Bloch procedures very challenging, if not prohibitive. Instead, we observe the proposed moiré phenomena by analyzing the out‐of‐plane displacement in real and reciprocal spaces for fixed frequency as a function of the twist angle.

Overall, we find evidence of a very rich behavior of the resulting metasurfaces, whereby different IFC transitions occur at different frequencies. We focus on the two hyperbolic regimes presented in Figure 1c. The first example at 485 Hz is illustrated in Figure 2b, at which the wave directionality rotates in the opposite direction compared to the twist angle, until *θ* = 30°. The effective wavelength of the guided waves drastically increases in the small angle regime ($\theta <10^{\circ }$), as displayed on Figure 2b, evidence of the progressive emergence of a moiré pattern introducing a super‐lattice with long spatial wavelengths (Figure 2a). We note that the metasurface response is strongly affected by the twist angle, as long as the wavelength of the moiré pattern is larger than the wavelength of the propagating waves. In reciprocal space, we correspondingly observe the presence of spatial harmonics that move away from the center of the Brillouin zone toward larger wavenumbers as the twist angle increases, in line with a decrease in moiré periodicity. When *θ* gets closer to $45^{\circ }$, an inverted phenomenon arises, albeit less noticeable in the 2D modulation profile, and some spatial harmonics move closer to the center, causing a distortion of the IFCs. Similar to the case at $\theta \;=4^{\circ }\;$, this effect hampers surface wave propagation, which is linked to the emergence of partial bandgaps and band flattening caused by the interaction of different spatial harmonics. The rigorous analysis of this phenomenon is inherently complex due to the quasiperiodic nature of the system and it goes beyond the scope of this work.

A different evolution of the supported band structure as a function of the twist angle can be observed in Figure 2c, corresponding to excitation at 625 Hz. At $\theta \;=0^{\circ }\;$ the wave propagation is hyperbolic but with opposite orientation. As *θ* increases, the field progressively loses directionality, and becomes completely delocalized above $10^{\circ }$. In reciprocal space (bottom row), this field evolution manifests itself as a topological transition of the associated IFCs, which evolve from open hyperbolic to closed elliptical for increasing twist angle. Similar to Figure 2b, this is explained by the distortion of the original contours due to emerging quasi‐periodic modulation and super‐lattice patterns. Although the transition here is driven by the twist angle, its emergence is inherently different from the ones observed in previous studies of twisted hyperbolic metasurface bilayers:^[^ ^32^, ^41^, ^42^, ^43^ ^]^ here the system consists of a single layer whose interleaved spatial modulation lattices and emerging moiré patterns directly control the coupling between resonators, governing the IFC features.

**Figure 3:**
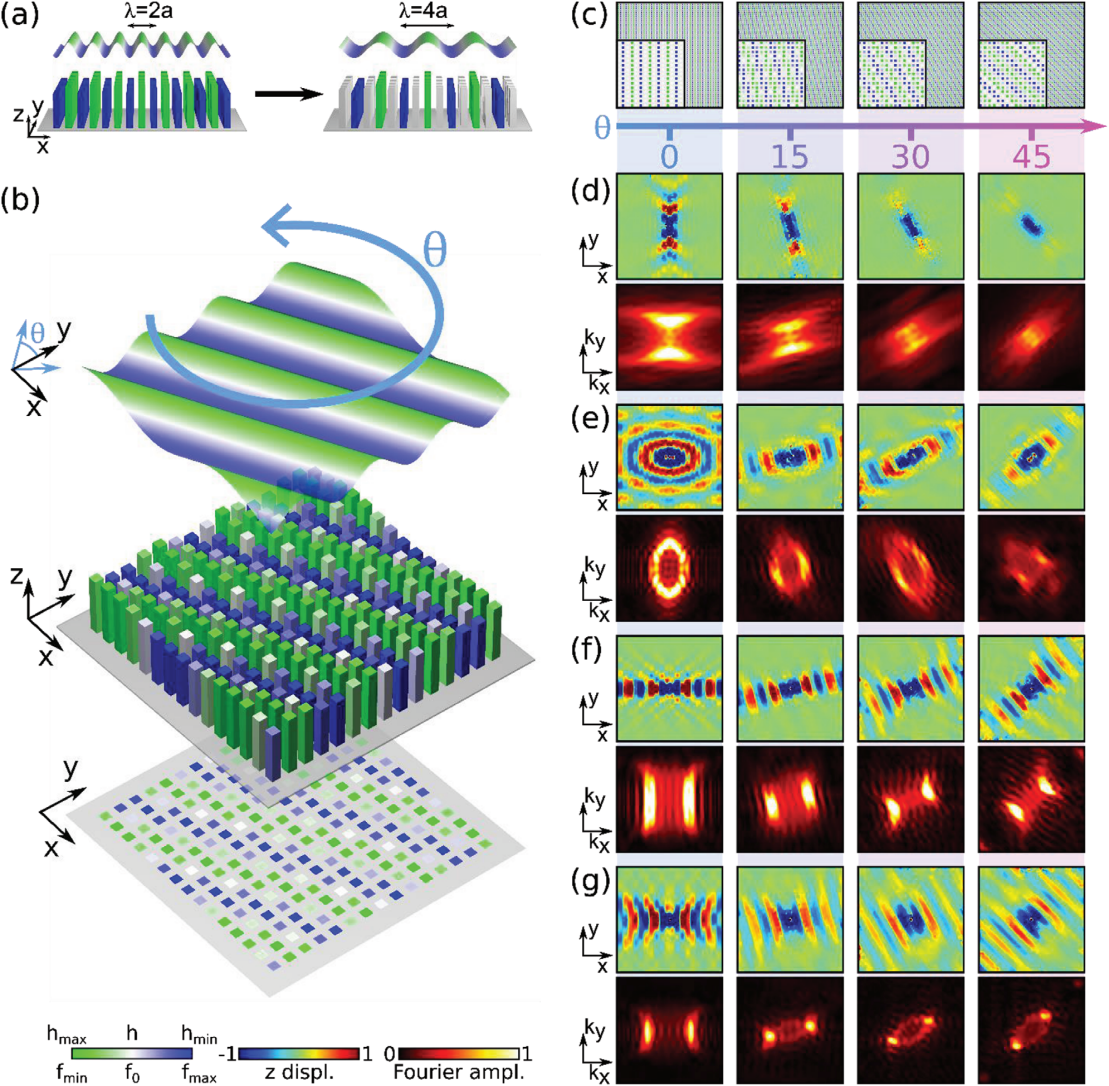
a) Doubling the modulation period enables a better sampling of the resulting modulation profile. b) The modulation profile is better preserved during the twist. c) Pillar height modulation as a function of rotation angle, with a zoom‐in inset for four angles. d) Simulated out‐of‐plane displacement field maps (top) and spatial FT (bottom) as a function of the rotation angle for hyperbolicity along *y* at 470 Hz. e–g) Same as (d) in the case of topological transitions at 595, 620, and 645 Hz, respectively.

Next, we explore the possibility of precisely tuning the dispersion profile across smoother topological transitions. As noted above, the spatial features emerging from twisting the two interleaved lattices are characterized by a large change in their periodicities as the twist angle is varied. Increasing the twist angle can quickly degrade the nature of the modulation, as a function of how coarse the height modulation is sampled by the period of the square lattice. The associated angular sensitivity of this phenomenon can be expectedly reduced by increasing the periodicity of the modulation profile. For example, **Figure** **3**a considers the untwisted scenario when the modulation period is doubled to *λ* = 4*a*. This change translates into a smoother correlation between twist angle and resulting anisotropic contours, with considerably smaller distortions (Figure 3b,c). As a consequence, the original spatially anisotropic distribution of couplings within the monolayer, which is responsible for the hyperbolic features, can be better preserved as the twist angle changes.

The resulting wave propagation features are summarized in Figure 3. Figure 3d considers the frequency for which the original untwisted structure supports directional hyperbolic waves oriented along the *y* axis, for excitation at 470 Hz. Although super‐lattice phenomena occur at small angles, their impact on the IFCs is less pronounced now, compared to Figure 2a. Indeed, as the angle increases, the propagation of directional waves smoothly follows the modulation rotation. In reciprocal space, the corresponding hyperbolic contours, albeit progressively flatter due to small changes in the couplings induced by moiré effects, undergo a similar rotation, indicating an effective twist of the metasurface properties occurring over a large angular range.

Next, we focus on the frequency range associated with hyperbolicity along the *x* direction, displayed on Figure 3e–g (for 595, 620, and at 645 Hz, respectively). In the untwisted case ($\theta \;=0^{\circ }\;$), these frequencies are related to different anisotropic phases: the contour in (e) is an ellipse that progressively opens as the frequency is increased to become flat in (f). A further frequency increase results in a curvature inversion, leading to a hyperbolic IFC (Figure 3g). As we increase the twist angle, Figure 3e demonstrates an opening of the IFC, and correspondingly a topological transition from elliptical to hyperbolic. The resulting canalized waves follow the rotation of the modulation profile until $\theta \;=45^{\circ }\;$. In the case of Figure 3f, an overall rotation of the flat contour, as well as its progressive curvature inversion, is observed as a function of the twist angle. Finally, Figure 3g shows a complete topological transition from hyperbolic to elliptical contours, driven by the twist. These findings clearly show that the moiré patterns induced by the twist between the interleaved lattices are responsible for topological transitions and canalized waves. The increased modulation wavelength (*λ* = 4*a*) results in a better preservation of the untwisted anisotropic coupling distribution. This smoothens the transitions compared to the results of Figure 2b and allows to observe these moiré phenomena over larger angular ranges.

**Figure 4:**
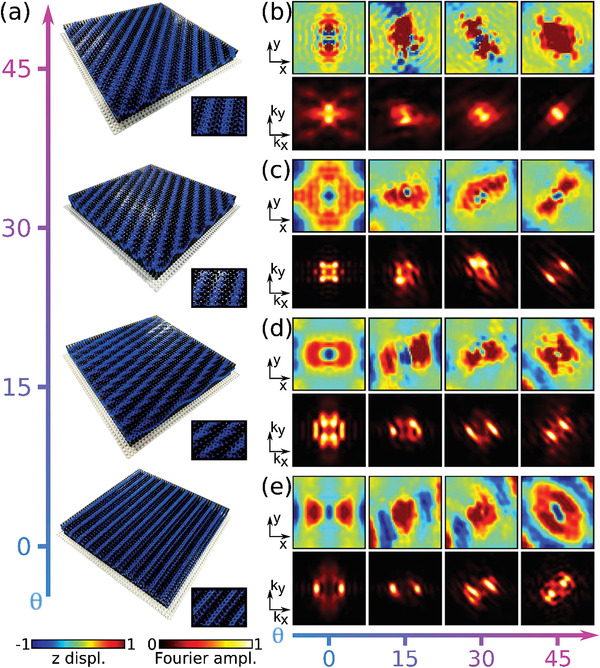
a) LEGO metasurfaces as a function of rotation angle for *λ* = 4*a*. b) Experimentally measured field maps (top) and spatial FT (bottom) as a function of the rotation angle in the case of hyperbolicity along the *y* axis at 311 Hz. c–e) Same as (b) in the case of topological transitions at 430, 452.5, and 462.5 Hz, respectively.

These results suggest a straightforward experimental implementation and observation of these phenomena on our reconfigurable LEGO platform comprising 44 × 44 resonators. We implemented several configurations for $\theta =0^{\circ }\;,\;15^{\circ },\;30^{\circ },\;45^{\circ }$, as shown in **Figure** **4**a. The sample snapshots illustrate the rotation of the modulation profile, as well as the distortion caused by the sampling as it is twisted relative to the interleaved metasurface lattice (see the pattern formed by the black and blue stripes in the insets). Figure 4b shows the experimentally measured field profile (top) and corresponding spatial FT (bottom) for hyperbolicity along *y* (at 311 Hz) as a function of the twist angle. As *θ* increases, the wave directionality accordingly rotates, reflected into the corresponding IFCs, which also become flatter and closer to the origin in Fourier space. This behavior, albeit less clear than in Figure 3d because of the smaller size of the sample, follows the trend seen in simulations. Figure 4c–e consider frequencies that support hyperbolic waves along *x*. Panel (c), for excitation at 430 Hz, starts from delocalized fields for $\theta \;=0^{\circ }\;$, and the propagation becomes strongly canalized as the angle increases, with a directionality following the modulation twist, consistent with Figure 3e. Our measurements confirm a moiré‐driven topological phase transition between closed and open contours. Next, Figure 4d, for excitation at 452.5 Hz, shows a rotation in wave directionality from $\theta \;=0^{\circ }\;$ to $45^{\circ }$. Although less evident due to the smaller size of the plate, this transition is consistent with the results in Figure 3f. Finally, Figure 4e presents results at 462.5 Hz, at which the waves are canalized and twisted from $\theta \;=0^{\circ }\;$ to $30^{\circ }$, and then become delocalized at $45^{\circ }$, experimentally confirming a reverse topological transition, from open to closed contours, as the twist angle increases, similar to Figure 3f. Overall, these experimental results clearly illustrate that tailoring the modulation parameters of twisted interleaved lattices over a metasurface produces topological transitions between delocalized and canalization regimes, as well as an effective rotation of the guided wave directionality, with the frequency being a key parameter that defines the type of observed transition.

Overall, these results showcase the rich behavior associated with moiré physics and hyperbolic dispersion within a single‐layer metasurface. We note that not all moiré physics associated to bilayer systems can be easily transposed to single layers. Notably, the interlayer coupling parameter is important for some applications, and it can be challenging to find its equivalent in single‐layer systems.^[^ ^45^, ^46^ ^]^ Moreover, while bilayers may be modeled based on the properties of the individual layers,^[^ ^32^, ^41^ ^]^ additional moiré effects and quasi‐periodicity are inevitable in our system, which makes their modeling more complex. Finally, while inducing dynamical reconfigurability in our single layer moiré system requires a more complex implementation of active devices, it does not rely on any physical displacement between the layers, making it more robust and fully controllable.

## 3 Conclusion

In this paper, we have investigated the effect of twisting interleaved lattices over a single‐layer pillared metasurface. We first explored the case where the two governing spatial features, the position and height modulation profile of the pillars, are aligned but feature a mismatch in their spatial period along one direction. The resulting periodic metasurface supports hyperbolic features over a broad range of frequencies, whose emergence has been observed both numerically and experimentally over a LEGO platform. Next, we introduced a relative rotation between the interleaved lattices, which induces moiré patterns that generates emerging wave phenomena, resulting in drastic modifications of the IFCs that undergo a transition from open to closed contours as the twist angle varies. A coarser sampling of the modulation patterns causes these transitions to be abrupt and to occur over a limited range of twist angles. The effect of sampling is expounded by increasing the modulation wavelength, which results in a better preservation of the spatial features as the twist angle changes, and produces smoother topological transitions. Such transitions are associated with extreme anisotropic features, inducing wave canalization along specific directions that are controlled by the twist angle within a range of angles and frequencies. In stark contrast to the case of twisted hyperbolic bilayers,^[^ ^32^, ^41^, ^42^, ^43^ ^]^ these transitions are driven by the moiré patterns emerging within the sample as the rotation angle changes. Moreover, albeit of topological nature, they differ from topological phases in chiral hyperbolic metamaterials, which are related to pseudo‐spin propagation at the edge of the system.^[^ ^62^ ^]^ We have observed these phenomena over a simple, practical, and highly reconfigurable LEGO platform, which allowed us to observe with flexibility the various regimes discovered in our study. As such and considering additional practical implementation challenges, they can be translated over a broad range of physical domains, including quantum and nanophotonic systems or microphononics in the context of pillared media,^[^ ^63^, ^64^ ^]^ opening opportunities for twistronic‐induced phenomena that do not require multilayered fabrication and careful control alignment, interlayer couplings, and twisting. The reconfigurability of our approach, in contrast with previously investigated twisted bilayer configurations, opens new opportunities for single‐layer moiré metasurfaces featuring high tunability of anisotropic responses. Such tunability emerges as a function of the twist angle, which is a single parameter defining the considered modulation. This suggests new opportunities stemming from the rich physics of twistronics and moiré phenomena, which may also open the door to dynamically reconfigurable devices capable of real‐time enhanced wave manipulation.

## Conflict of Interest

The authors declare no conflict of interest.

## Supporting information

Supporting Information

## Data Availability

The data that support the findings of this study are available from the corresponding authors upon reasonable request.
