# Supplementary material for: Moiré‐Driven Topological Transitions and Extreme Anisotropy in Elastic Metasurfaces
Source: Adv Sci (Weinh). 2022 Mar 6;9(13):2200181. doi: 10.1002/advs.202200181 (PMC9069188; doi:10.1002/advs.202200181)
Supplement: Supplementary file 1 — Supporting Information [file ADVS-9-2200181-s001.pdf]

ADVANCED SCIENCE
Open Access

Supporting Information

for *Adv. Sci.*, DOI 10.1002/advs.202200181

Moiré-Driven Topological Transitions and Extreme Anisotropy in Elastic Metasurfaces

Simon Yves, Matheus Inguaggiato Nora Rosa, Yuning Guo, Mohit Gupta, Massimo Ruzzene and Andrea Alù**

Supporting Information

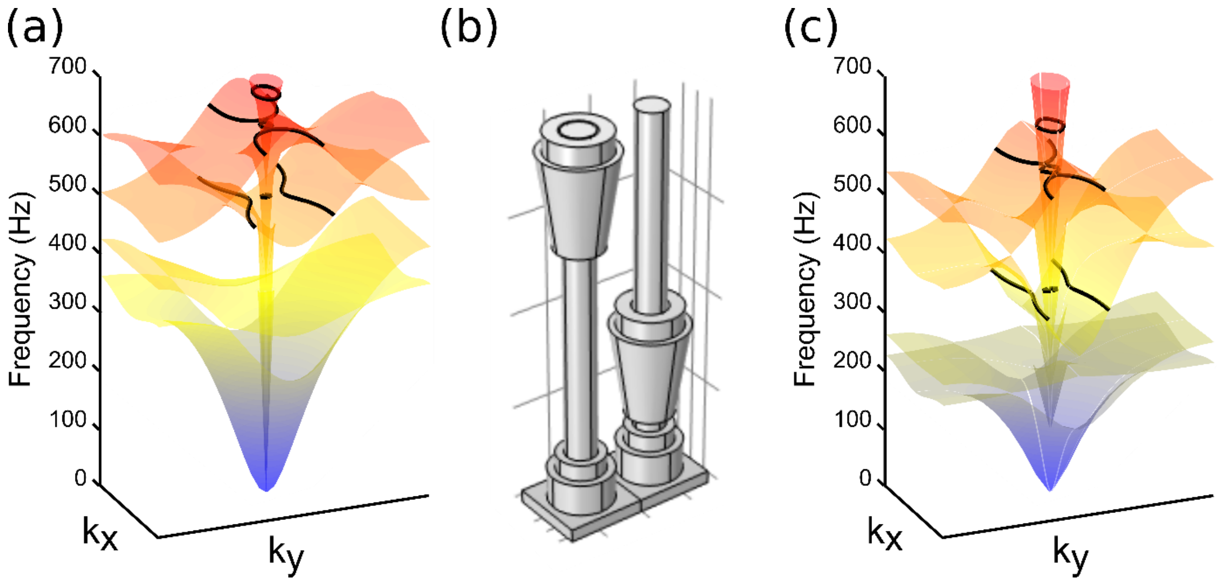

Figure S1. a) Band structure computed with pillars. b) COMSOL model of the LEGO unit cell. c) Band structure computed with (b).

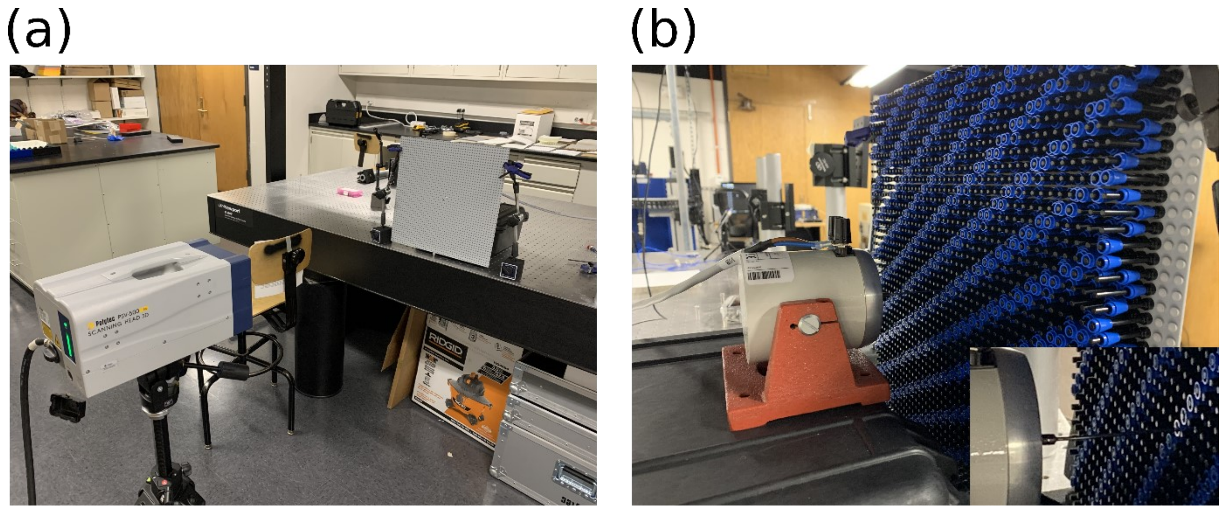

Figure S2. a) Experimental set up. b) Excitation of the sample.

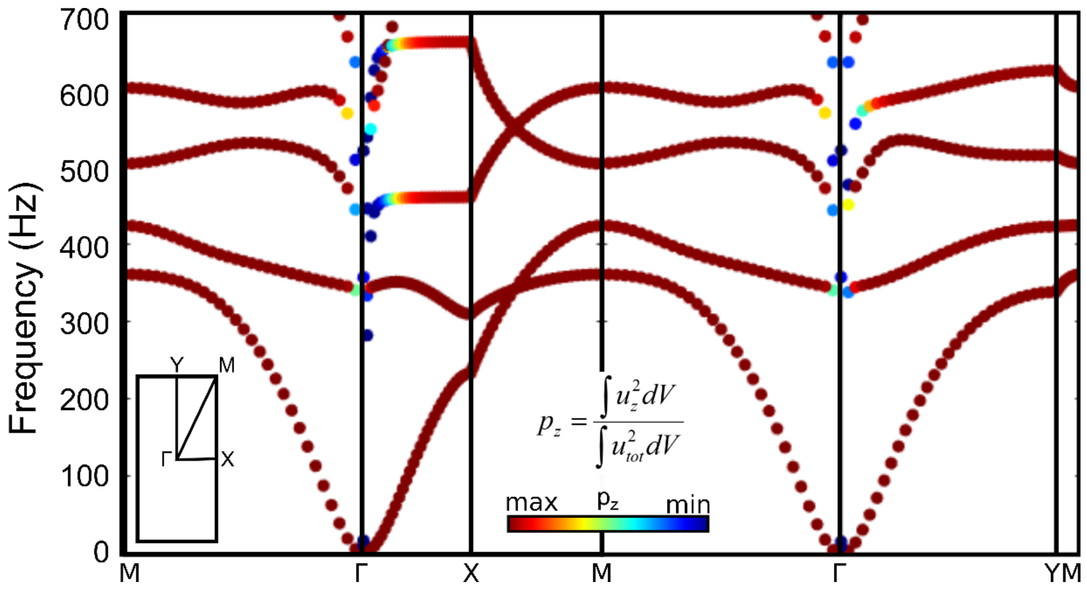

Figure S3. Dispersion relation of the pillars with the corresponding mode out-of-plane polarization p_z.

Section S1: Simulation Methods

The simulations results presented in this paper have been obtained with COMSOL Multiphysics Solid Mechanics with the following parameters: plate thickness $h_p = 1.3$ mm, step of the lattice $a = 11.31$ mm, width of the pillar $c = 4$ mm, mean height of the pillars $h_0 = 36$ mm, modulation amplitude $\alpha = 0.1$. The material of the plate and pillars is polyactic acid (PLA, Young modulus $E = 2.5$ GPa, Poisson ratio η=0.3, density $\rho = 1{,}3$ gcm^{-3}). The twist angle θ goes from 0° to 45°, the behavior of the system being inverted between 45° and 90°. The band structure of the untwisted case is obtained by applying Bloch periodic boundary conditions to the sides of the unit cell in the eigenfrequency module. It contains four bands in the case presented in Fig.1, and we focus on the two high frequency bands where the hyperbolicity is clearer and more broadband.

The results related to samples of finite size are obtained thanks to the frequency response module. Perfectly Matched Layers (PML) are displayed around the 80x80 unit cell sample in order to limit the reflections. An out-of-plane displacement source is placed at the center of the system, on the bottom size of the plate, in order to excite efficiently the transverse Lamb waves in the plate. At each operating frequency, the corresponding out-of-plane displacement field maps on the bottom side of the plate is recorded and a 2D spatial Fourier transform is applied to highlight the corresponding IFC.

An accurate simulation of the LEGO medium is provided as an Extended Data Figure. The comparison between the latter and the pillars result shows an overall good agreement in the shape of the band structures, the physics of the system being governed by the bending nature of the resonant inclusions. We used the pillars to illustrate our points through the paper as their simpler geometry allows faster computation.
